# Supplementary material for: Overcoming TRAIL-resistance by sensitizing prostate cancer 3D spheroids with taxanes
Source: PLoS One. 2021 Mar 4;16(3):e0246733. doi: 10.1371/journal.pone.0246733 (PMC7932526; doi:10.1371/journal.pone.0246733)

DU145 Cells
1 - Untreated 2D
2 - Untreated 3D
3 - 0.25μM CBZ 2D
4 - 0.25μM CBZ 3D
5 - 0.25μM DTX 2D
6 - 0.25μM DTX 3D

PC3 Cells
7 - Untreated 2D
8 - Untreated 3D
9 - 0.25μM CBZ 2D
10 - 0.25μM CBZ 3D
11 - 0.25μM DTX 2D
12 - 0.25μM DTX 3D

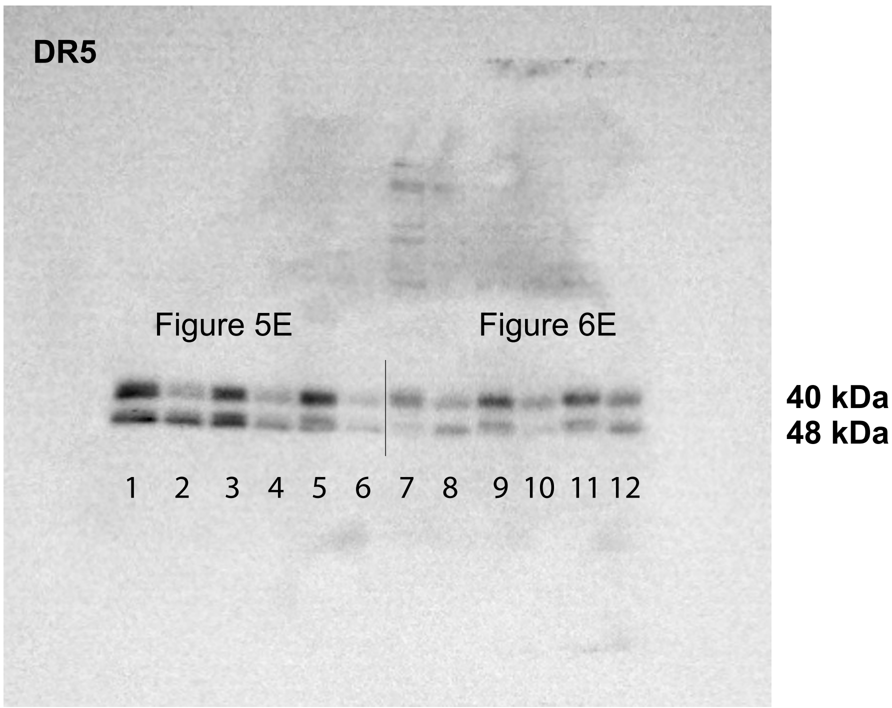

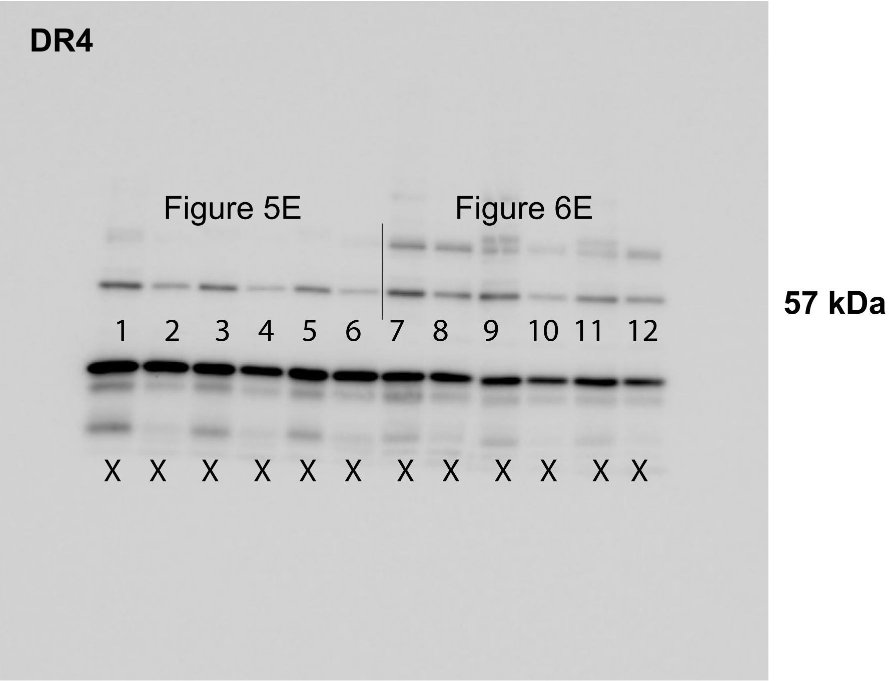

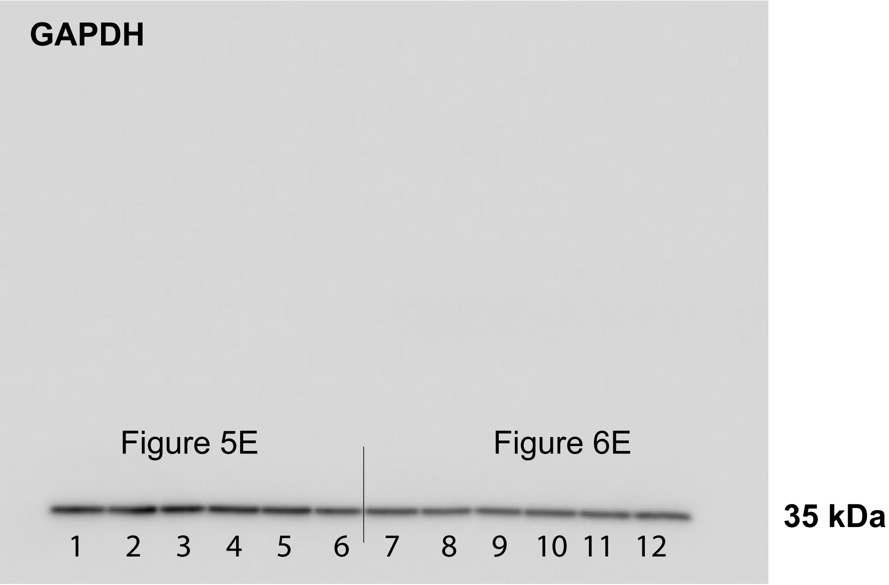

Supplement: S1 Fig — (PDF) [file pone.0246733.s001.pdf]
