# Supplementary material for: Overcoming TRAIL-resistance by sensitizing prostate cancer 3D spheroids with taxanes
Source: PLoS One. 2021 Mar 4;16(3):e0246733. doi: 10.1371/journal.pone.0246733 (PMC7932526; doi:10.1371/journal.pone.0246733)
Supplement: S2 Fig — (PDF) [file pone.0246733.s002.pdf]

Mycoplasma Test Results via Universal Mycoplasma Detection Kit (ATCC 30-1012K)

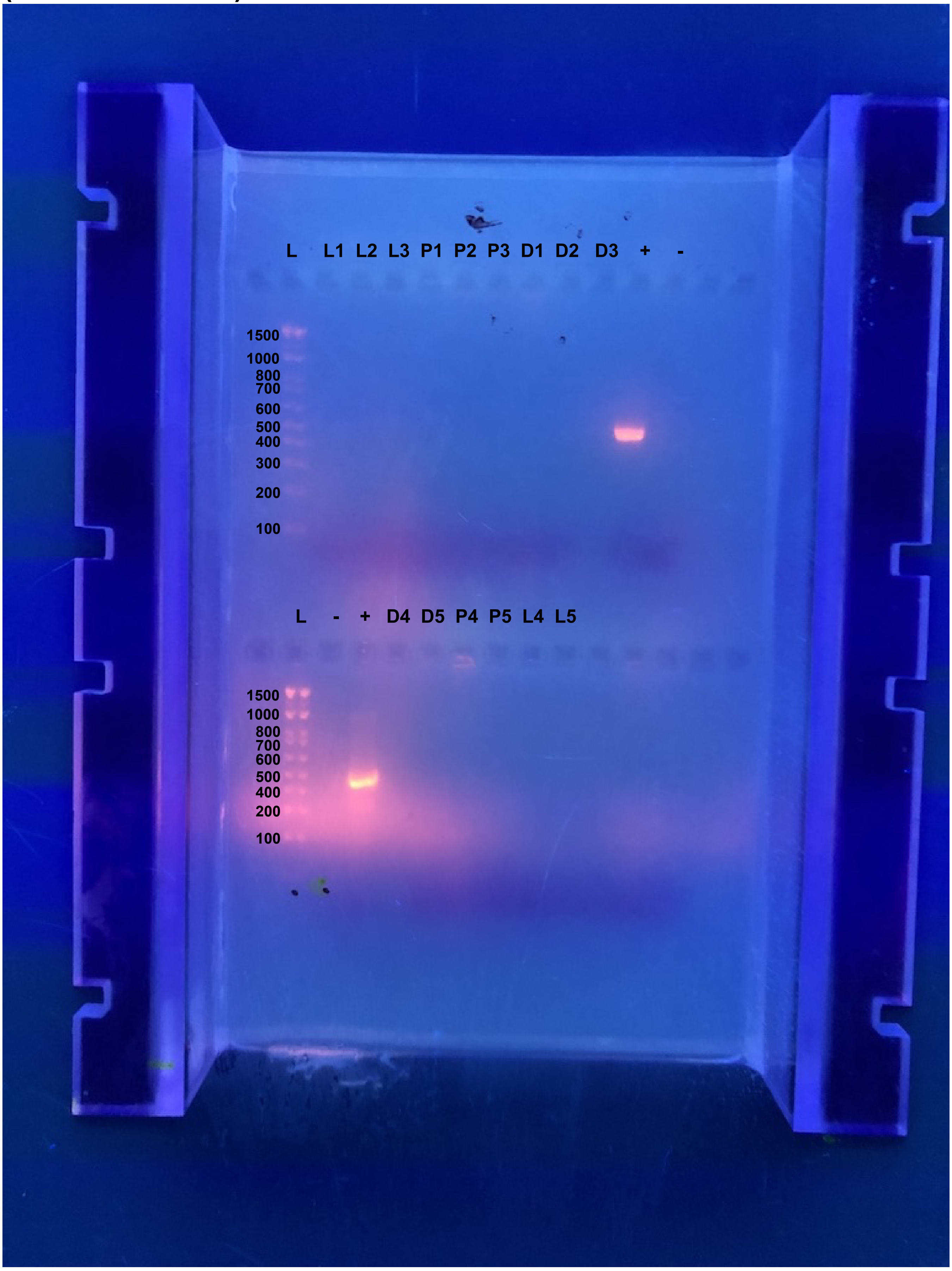

L: Ladder (-): Negative Control (+): Positive Control

L1 - LNCaP Passage 2
L2 - LNCaP Passage 2
L3 - LNCaP Passage 2
L4 - LNCaP Passage 8
L5 - LNCaP Passage 8

P1 - PC3 Passage 3
P2 - PC3 Passage 3
P3 - PC3 Passage 3
P4 - PC3 Passage 15
P5 - PC3 Passage 15

D1 - DU145 Passage 3
D2 - DU145 Passage 3
D3 - DU145 Passage 3
D4 - DU145 Passage 15
D5 - DU145 Passage 15
